# Editors’ Medal 2026

**DOI:** 10.1186/s42155-026-00739-y

**Published:** 2026-07-15

**Authors:** Robert A. Morgan

**Affiliations:** St George’s University Hospitals NHS Foundation Trust and City St Georges, London, UK

As I approach the 12-month anniversary of my installation as Editor-in-Chief of CVIR Endovascular, it gives me immense pleasure to announce the recipient of the 2026 CVIR Endovascular Editor’s Medal. Before I do that, I would like to say a few words about the progress of CVIR Endovascular in the last 12 or so months.

In 2025, CVIR Endovascular published 122 articles, which are all available free for open access on the CVIR Endovascular website. This compares with 92 articles published in 2024 and 63 articles published in 2023. The breakdown of published articles is also changing with increased original articles (47% of original articles accepted in 2025 vs 35% accepted in 2023), and a reduction in the number of case reports (23% in 2025 vs 31% in 2023). This indicates the ongoing maturity of the journal with acceptances and publication of increasingly higher quality articles year on year. This evolution in quality has been recognised by the recent increase in impact factor (IF) to 2.1 in 2025 from 1.5 in 2024 and the initial impact factor of 1.2 in 2023. The increase in IF to 2.1 is in large part due to the sterling work of the previous Editor-in-Chief, Jim Reekers, who I am thankful to for so expertly helming the journal from its inception to August 2025.

Now to the subject of this Editorial, namely the 2026 Editors’ Medal. This award is made for the best published article in 2025. For the first time, the awardees will be presented with their medal during the opening ceremony at the 2026 CIRSE congress in Copenhagen this September.

How did we decide on the winner? The Section and Regional Editors for the journal who work tirelessly processing the articles that you submit were asked to name their top articles published in 2025, based on academic quality, readability, citations and downloads. From the top 10 articles, the editors were asked to vote for their preferred choice. From the 122 articles published in 2025, I am pleased to inform you that the winner of the 2026 CVIR Endovascular Editor’s medal is “A fifteen‑year retrospective analysis of varicocele embolization: evaluating success, recurrence rates and embolic agents [1]”, authored by Meadhbh Ni Mhiochain de Grae, Maha Al‑khattab, Amor Alkadhimi, Maia Springael and Gerry O’Sullivan from the Interventional Radiology departments at Galway University Hospital, Galway and Tallaght University Hospital, Dublin, in Ireland. This article is a retrospective review of 225 patients who underwent embolization for varicoceles in two tertiary centres between 2008 and 2023. The authors reported technical and clinical success rates in 96% and 93.75% respectively and conclude that their findings support the role of embolization as first-line treatment in varicocele management.

Other articles worthy of special mention in 2025 were as follows: “Postoperative symptom changes following uterine artery embolization for uterine fibroid based on FIGO classification [2]” by Nozaki et al., “N-butyl cyanoacrylate glue application in prostate artery embolization for benign prostatic hyperplasia: a systematic review of safety and efficacy [3]” by Mohamed et al., “Contemporary carotid artery stenting practices and peri-procedural outcomes in different European countries: ROADSAVER study multicentric insights [4]” by Muller-Hulsbeck et al., “Percutaneous Closure Device Controlled INCRAFT Stentgraft Implantation Registry (PUCCINI) [5]” by Engelen et al., “A multicenter prospective study evaluating use of EmboCube™ Embolization Gelatin alone or in combination with other embolic agents to control bleeding [6]” by Pellerin et al., and “Prospective analysis of bleomycin electrosclerotherapy for clinical outcome and volume reduction in therapy refractory slow-flow malformations [7]” by Deleu et al. All the above articles are deserving of your attention, and I am pleased to commend them to you.

In summary, the winner of the 2026 CVIR Endovascular Editors’ Medal is “A fifteen‑year retrospective analysis of varicocele embolization: evaluating success, recurrence rates and embolic agents.”, authored by O’Sullivan et al. I look forward to seeing as many of you as possible for the medal presentation at Copenhagen in September.

## Robert Morgan

Editor-in-Chief CVIR Endovascular.

## Data Availability

Not applicable
